# Unveiling the Dynamic Interplay between Cancer Stem Cells and the Tumor Microenvironment in Melanoma: Implications for Novel Therapeutic Strategies

**DOI:** 10.3390/cancers16162861

**Published:** 2024-08-16

**Authors:** Patrizia Limonta, Raffaella Chiaramonte, Lavinia Casati

**Affiliations:** 1 Department of Pharmacological and Biomolecular Sciences “R. Paoletti”, Università degli Studi di Milano, 20133 Milan, Italy; 2 Department of Health Sciences, Università degli Studi di Milano, 20142 Milan, Italy; raffaella.chiaramonte@unimi.it

**Keywords:** melanoma, cancer stem cells (CSCs), CSC markers, tumor microenvironment (TME), CSC-TME interplay, bidirectional cell-to-cell communication, CSC-targeted therapies

## Abstract

**Simple Summary:**

Resistance to standard therapies represents a major challenge in melanoma treatment. This is mainly related to the high heterogeneity of this malignancy due to the presence of different types of cells in the tumor mass. Cancer stem cells (CSCs) are a small subset of cells endowed with self-renewal and tumorigenic capacity and the ability to escape anticancer therapies. Melanoma CSCs can be identified by the expression of biomarkers and intracellular pathways deeply involved in their malignant phenotype. Dissecting the molecular mechanisms underlying CSC biological functions might pave the way for novel therapeutic approaches. Herein, we highlight the intricate bidirectional communication between melanoma CSCs and their surrounding cells in the tumor microenvironment and discuss how this interplay might impact the processes of drug resistance and tumor relapse. We also address the potential benefits of CSC-targeted strategies, in combination with conventional drugs, for improving melanoma treatment strategies.

**Abstract:**

Cutaneous melanoma still represents a significant health burden worldwide, being responsible for the majority of skin cancer deaths. Key advances in therapeutic strategies have significantly improved patient outcomes; however, most patients experience drug resistance and tumor relapse. Cancer stem cells (CSCs) are a small subpopulation of cells in different tumors, including melanoma, endowed with distinctive capacities of self-renewal and differentiation into bulk tumor cells. Melanoma CSCs are characterized by the expression of specific biomarkers and intracellular pathways; moreover, they play a pivotal role in tumor onset, progression and drug resistance. In recent years, great efforts have been made to dissect the molecular mechanisms underlying the protumor activities of melanoma CSCs to provide the basis for novel CSC-targeted therapies. Herein, we highlight the intricate crosstalk between melanoma CSCs and bystander cells in the tumor microenvironment (TME), including immune cells, endothelial cells and cancer-associated fibroblasts (CAFs), and its role in melanoma progression. Specifically, we discuss the peculiar capacities of melanoma CSCs to escape the host immune surveillance, to recruit immunosuppressive cells and to educate immune cells toward an immunosuppressive and protumor phenotype. We also address currently investigated CSC-targeted strategies that could pave the way for new promising therapeutic approaches for melanoma care.

## 1. Introduction

Malignant melanoma still represents the most serious type of skin cancer worldwide and the deadliest form of cutaneous tumors, accounting for up to 80% of cancer-related deaths among skin cancer patients [1]. Most melanomas are diagnosed in the early stage and can be effectively treated with surgery; on the other hand, late-stage tumors undergoing dissemination and metastasis are associated with a very poor prognosis [2,3].

In recent decades, gene mutation analysis has been increasingly utilized to improve diagnosis, classification, prognosis and risk stratification in different tumors, paving the way for targeted therapeutic interventions [4,5,6,7]. In melanoma, driver mutations of the BRAF and NRAS oncogenes are detected in about 40–50% and 10–20% of patients, respectively, leading to the overactivation of the mitogen-activated protein kinase (MAPK) signaling pathway (RAS/RAF/MEK) together with the parallel PI3K/Akt/mTOR cascade, both deeply involved in the processes of cell growth and survival. Additional driver mutations have been found to involve the neurofibromin 1 (NF1) and the KIT (1–2%) genes [8,9,10,11], while mutations associated with tumor development have been observed in the cyclin-dependent kinase inhibitor 2A (CDKN2A) gene, a well-known cell cycle regulator [12]. These observations led to the development of “targeted therapies” based on BRAF inhibitors (vemurafenib, dabrafenib, encorafenib) or MEK inhibitors (trametinib, cobimetinib, binimetinib), either alone or in combination; however, despite the high response rate and reduced toxicity of the combination treatment compared with the use of single compounds, most patients experience a low survival rate (5 years) and drug resistance within a few months [13,14,15,16,17,18,19].

Immunotherapy has emerged as a novel promising therapeutic strategy to trigger antitumoral immunity. In this context, immune checkpoint inhibitors were developed with the aim to improve the activity of the antitumor immune system. Specifically, the inhibitors of the lymphocyte-activation gene 3 (LAG3) transmembrane protein (relatlimab), the programmed cell death-1 (PD-1) receptor (nivolumab, pembrolizumab), the PD-1 ligand (PD-L1) (atezolizumab) and the cytotoxic T-lymphocyte antigen 4 (CTLA-4) (ipilimumab and tremelimumab) have been approved by the Food and Drug Administration (FDA) and are presently used in clinical practice. These drugs, either alone or in combination, have drastically improved melanoma clinical management; however, some limitations still remain due to their association with serious side effects and to the development of primary and acquired resistance [17,18,19,20,21,22,23,24].

The mechanisms underlying the development of resistance to standard-of-care treatments in melanoma have been extensively investigated in the past few years. In patients undergoing targeted therapy treatments, primary (intrinsic) resistance has been reported to be associated with the aberrant activation (ligand-induced dimerization or oligomerization) of receptor tyrosine kinases (RTKs), which in turn trigger a cascade of phosphorylation and, ultimately, the activation of the RAS/RAF/MEK/ERK and PI3K/Akt/mTOR pathways. Phosphatase and tensin homolog (PTEN) is a protein endowed with a peculiar tumor suppressive activity and the most important negative regulator of the PI3K/Akt oncogenic pathway. The increase in PI3K/Akt/mTOR signaling occurring in drug resistance may also be induced by the deletion or loss-of-function mutations of PTEN.

Acquired resistance, developing in initial responders to therapies, mostly results from the reactivation of the MAPK pathway and is associated with alterations in NRAS, BRAF, MEK and NF1 [25,26,27,28,29,30,31,32,33,34]. The RAF isoforms CRAF and ARAF are also frequently dysregulated, allowing mutated BRAF to be bypassed and leading to the reactivation of ERK [35,36,37]. Moreover, the Cancer Osaka thyroid (COT) kinase has been reported to be overexpressed in drug-resistant melanomas and to induce resistance to BRAF inhibition through the direct activation of the MEK/ERK signaling pathway [38]. Additional mechanisms involved in the development of acquired resistance include amplification or alternative splicing of the BRAF gene, alterations in miRNA expression, cell phenotype plasticity defined by the expression of the microphthalmia-associated transcription factor (MITF), activation of different signaling cascades such as the HIPPO/YAP/TAZ pathway and epigenetic regulation of DNA accessibility [39,40,41,42,43,44,45,46,47,48]. Primary and acquired resistance have also been reported to occur in patients challenged with immune checkpoint inhibitor therapies and found to be associated with different mechanisms: poor immunogenicity, impaired maturation of dendritic cells (DCs), altered expression levels of the immunosuppressive marker PD-L1, insufficient T cell activation, antigen-presenting cell dysfunction, loss-of-function mutations in genes involved in antitumor signals (interferon-γ, IFN-γ), melanoma cell dedifferentiation and phenotypic plasticity [20,23,47,49,50,51].

Melanoma is a tumor characterized by high heterogeneity and transcriptional plasticity [52,53,54]. Two models of carcinogenesis have been proposed to explain this heterogeneity. According to the stochastic model, all the tumor cells are biologically equivalent, with the same tumorigenic ability, and acquire different characteristics depending on intrinsic factors, such as genetic and epigenetic alterations and intracellular signaling pathways, and extrinsic factors from the tumor microenvironment (TME). In the hierarchical model, a tumor originates from a small subpopulation of slow-cycling “cancer stem cells” (CSCs) that reside at the top of the hierarchy. These cells acquire mutations but, like normal stem cells, show self-renewal ability as well as the potential to give rise to different progeny cells responsible for the formation of the tumor bulk population through asymmetric cell division [55,56,57,58]. This hierarchical model has been widely considered the most common model of tumor development. However, the recently identified high level of plasticity of tumor cells reconciles the two models and suggests that they may not be mutually exclusive, based on the observation that differentiated cancer cells can shift to a stem cell phenotype and, conversely, stem cells can be converted again into a non-stem cell state [59,60]. In line with these data, Quintana and coworkers reported that 25% of unselected patient-derived melanoma cells could generate the bulk tumor mass when inoculated in small numbers in nude mice. Since this percentage is higher than the small subpopulation of CSCs located into the tumor bulk, these authors conclude that also non-classical CSCs could generate tumors when inoculated in nude mice [61].

Growing bodies of evidence support that CSCs play key roles in tumor progression, treatment resistance and relapse in different tumors, such as malignant melanoma [34,62,63,64,65,66]. Notably, a reciprocal interplay has been observed between CSCs and their TME, which maintains or even promotes CSC survival and self-renewal, thus participating in tumor promotion, resistance and recurrence [67,68,69,70,71]. Based on these considerations, eradication of CSCs is presently considered a potentially promising strategy to overcome drug resistance in cancer. A deeper understanding of the molecular features and biological functions of CSCs might improve the therapeutical options for aggressive tumors, such as melanoma.

Herein, we provide an overview of the molecular and functional characteristics of melanoma CSCs, such as cell immunophenotypical markers, activated signaling pathways and biological roles in melanoma development and progression; the dynamic interplay between melanoma CSCs and their TME will be specifically discussed to shed light on how this interaction might impact tumor progression, resistance and recurrence. We also address currently investigated CSC-targeted strategies that could improve the therapeutic approaches for melanoma care.

## 2. Melanoma CSC Markers

The interest in the identification of melanoma CSCs prompted the search for specific markers. In vitro, CSCs can grow when cultured in low-adherence cell culture conditions in the presence of growth factors, such as fibroblast growth factor-2 (FGF-2) and epidermal growth factor (EGF) generating floating spheroid colonies (melanospheres) and express markers of epithelial-to-mesenchymal transition (EMT) associated with a highly invasive behavior; in vivo, the inoculation of CSCs in nude mice at limited dilutions has been widely reported to give rise to newly formed tumors sharing the characteristics of the primary tumor mass [63,65,72,73,74,75,76,77].

**Figure 1:**
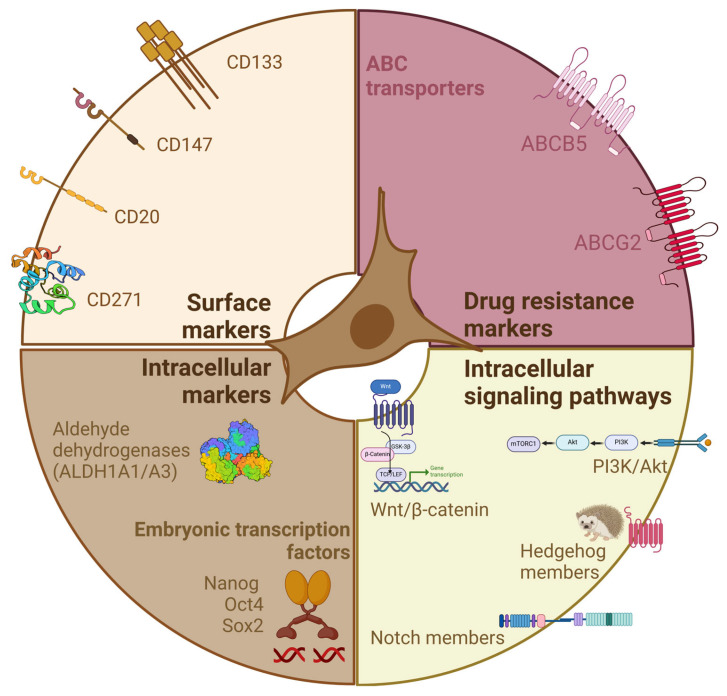
Schematic representation of stemness biomarkers and intracellular pathways in melanoma CSCs. ABC, ATP-binding cassette transporter.

In addition, melanoma CSCs may be identified by the expression of specific stemness biomarkers, such as surface markers associated with melanocyte undifferentiated state or with drug resistance (ATP-binding cassette transporters involved in drug efflux out of the cells) and intracellular markers involved in drug metabolism (i.e., those belonging to the aldehyde dehydrogenase -ALDH- family). Embryonic stem cell markers, such as Sox2, Oct4 and Notch, have also been shown to be overexpressed in melanoma CSCs (Figure 1).

However, the specificity of most of these markers for CSCs remains a serious controversial issue. By means of in vitro and in vivo experiments, it has been shown that they are expressed in a large population of melanoma cells, being not limited to the CSC subpopulation, as well as in differentiated non-tumoral cells, such as normal melanocytes [63,77,78,79,80]. Alterations in specific intracellular signaling pathways have also been found to correlate with melanoma cell stemness (Figure 1).

### 2.1. Surface Markers

CD133 (prominin-1/AC133), a member of the pentaspan transmembrane glycoproteins, is considered a relevant surface marker for the identification of CSCs in different types of solid tumors. Specifically, high levels of CD133 were reported to be present in melanoma tissue biopsies when compared to nevi and to correlate with tumor growth [81,82]. In line with this observation, CD133+ melanoma cells are endowed with a high tumorigenic potential when inoculated in nude mice and display in vitro a peculiar metastatic behavior [82,83,84,85,86]. Moreover, CD133-dependent mechanisms are deeply involved in the development of melanoma resistance to standard therapies [34,64]. Mechanistically, the key role of CD133 in maintenance of stemness properties and drug resistance has been demonstrated to be mediated by the activation of the PI3K/Akt/mTOR intracellular signaling pathway [34,64,87]. However, in contrast to these observations, the suitability of CD133 as an appropriate marker for identifying and selecting melanoma CSCs is still a matter of debate [88,89,90]. Since CD133 expression is not limited to the surface of CSCs, distinct biological functions of this protein have been pointed out in cancer cells [91].

CD271 (p75 neurotrophin receptor), the low-affinity nerve growth factor receptor, is a member of the tumor necrosis factor receptor family: it is well known to be expressed in the neural crest throughout embryonic development. High expression levels of CD271 have also been widely detected in melanoma cells and shown to positively correlate with high tumorigenicity, metastatic behavior and drug resistance [92,93,94,95,96,97,98]. In line with these observations, CD271 has been shown to regulate phenotype switching of melanoma cells from a non-stem to a stem-like state [99,100,101,102]. However, also in this case, the reliability of this surface receptor as a suitable melanoma CSC marker is still controversial [59,63,78,80,103,104].

CD147, a transmembrane glycoprotein belonging to the immunoglobulin superfamily, has been reported to play a key role in melanoma initiation and progression toward its aggressive metastatic stage [105,106,107]. Very recently, Jiang and coworkers demonstrated that melanoma cells expressing high levels of CD147 are endowed with stemness features, such as strong sphere formation ability, migration, invasion and high tumorigenic capacity when inoculated into nude mice. Interestingly, the elevated expression of this marker is associated with the activation of the TGF-β1 (transforming growth factor-beta 1) and Notch1 (neurogenic locus notch homolog protein 1) intracellular signaling pathways [108]. However, as stated by these authors, additional studies are necessary to confirm the potential role of CD147 as a novel marker in melanoma treatments.

CD20 is a cell surface protein normally expressed on B cell surface. CD20 has been reported to be consistently expressed on melanoma CSCs and to positively correlate with stemness-related markers [83]. Moreover, it has also been identified in melanoma patient specimens [109].

### 2.2. Markers of Drug Resistance

The ATP-binding cassette (ABC) transporters are a superfamily of proteins widely known to facilitate the ATP-driven efflux of drugs across cellular membranes, being deeply involved in drug resistance [110,111]. Based on this, their presence has been evaluated at the plasma membrane level in different tumors, including melanoma, and specifically in their CSC subpopulation [63,84,112,113,114,115,116]. ABCB5 has been proposed as a reliable marker of melanoma aggressiveness, multidrug resistance and stemness [84,117,118,119]; it has also been found to induce peculiar modifications of the cellular metabolism in melanoma-initiating cell lines [120]. Interestingly, ABCB5 exists in two isoforms: ABCG5FL, a full transporter, and ABCB5β, a half transporter whose homodimer does not confer multidrug resistance. Very recently, Gerard et al. identified two novel heterodimers of ABCB5β in melanoma cell lines, ABCB5β/B6 and ABCB5β/B9, endowed with ATPase activity. However, as suggested by these authors, the physiological functions of these dimers in melanoma progression still remain to be investigated [121].

In contrast to these observations, Louphrasitthiphol et al. suggested that ABCB5 unlikely represents a marker of melanoma CSCs since its expression is also associated with melanoma cells exhibiting markers of differentiation. Specifically, MITF, the transcription factor known to sustain the expression of ABCB5, is highly expressed in differentiated melanoma cells and this correlates with high levels of ABCB5 [122]. Moreover, a low MITF/high Axl (a receptor tyrosine kinase) ratio was reported in a subpopulation of slowly proliferating melanoma cells endowed with invasive properties and drug resistance [123]. Thus, the low/high MITF ratio could have an essential role in the switch between dedifferentiated and differentiated melanoma cells. Based on these observations, further studies are needed to confirm the reliability of the MITF/ABCB5 axis as a marker of melanoma cell stemness.

ABCG2 is another ABC drug efflux transporter associated with drug resistance and stemness in different types of tumors, such as melanoma [82,124,125]. ABCG2 was found to be highly expressed, together with other stemness markers, in melanoma tissues and to correlate with the clinical features of patients and a worse prognosis [126]. Moreover, it has been shown that melanoma cells resistant to vemurafenib express high levels of ABCG2 and CD271 [127]. Interestingly, Miranda-Lorenzo and coworkers reported that, in epithelial cancer cells, the subpopulation of cells showing CSC traits is endowed with high autofluorescence levels. Specifically, this autofluorescence was shown to be related to the presence of vitamin B2 (riboflavin) in membrane-located cytoplasmic vesicles coated with ABCG2, supporting the hypothesis that autofluorescence might represent a reliable marker of cancer cell stemness [128]. In line with these observations, in our laboratory, we could demonstrate that melanoma cells endowed with autofluorescence properties and expressing high levels of ABCG2 are able to form tumorspheres, express intracellular stemness markers and generate the bulk tumor mass when inoculated at low dilutions in nude mice [78].

### 2.3. Intracellular Markers

CSCs can also be identified by the overexpression of markers located at the intracellular level, such as detoxifying enzymes and pluripotency transcription factors.

ALDH are enzymes critical for detoxifying different endogenous and exogenous substrates by oxidizing aldehydes to corresponding carboxylic acids. ALDH1 (ALDH isozyme 1), specifically the isoforms ALDH1A1 and ALDH1A3, have been reported to be highly expressed and even overactivated in melanoma CSCs and to correlate with different biological properties of tumor-initiating cells, such as tumorigenesis and drug resistance [129,130]. In line with these observations, Sarvi et al. demonstrated that, in melanoma-initiating cells, the anticancer compounds 5-nitrofurans could be metabolized by ALDH1A1/1A3 into their bioactive, cytotoxic metabolites, which in turn effectively eradicate the subpopulation of melanoma cells, which expresses high levels of ALDH1 [131]. Pharmacological approaches targeting ALDH isozymes are currently being explored with the aim of specifically targeting and eliminating CSCs in different tumors [132,133].

Accumulating evidence supports that embryonic transcription factors (Sox2, Oct4, Nanog) are present in melanoma CSCs where they work in concert to maintain cell stemness; moreover, their expression levels correlate with cell stemness features, such as pluripotency, tumor-initiating capacity and drug resistance [134,135,136,137,138,139]. Sox2 has been reported to positively regulate the self-renewal of cells in melanoma spheroids as well as of ALDH+ melanoma cells [140]. In melanoma cells, this transcription factor was also shown to promote a CSC-like phenotype associated with resistance to chemotherapy or targeted therapy by transactivating the expression of the CD24 and ABCC1 stem cell markers [141,142]. Similar observations were reported for the Oct4 transcription factor. Specifically, Cordaro and coworkers reported that melanoma cells made resistant to dabrafenib exhibit peculiar stem cell-like traits accompanied by the translocation of Oct4 from the cytoplasm into the nucleus and the increased expression of the stemness marker CD20 [143]. Oct4 expression was also found to correlate with worse prognosis and survival in melanoma tissues, suggesting that it might represent a useful biomarker in melanoma prognosis [144]. Last but not least, Nanog overexpression was observed to boost the metastatic potential of melanoma cells through the upregulation of genes involved in glucose transport and oxidative phosphorylation (OXPHOS) [145] and to promote immune resistance and stem-like properties in immune-refractory tumor cell types, including melanoma cells [146]. In contrast to this evidence, Khoo and colleagues recently reported that Nanog-overexpressing melanoma cells release small extracellular vesicles (EVs) endowed with a striking metastasis-suppressive behavior if compared to EVs derived from the original melanoma cell line [147]. Therefore, further studies are needed to establish the role of the Nanog transcription factor as a pluripotency marker in melanoma.

### 2.4. Intracellular Signaling Pathways

It is now well established that different developmental signaling pathways, particularly Wnt/β-catenin, Notch, Hedgehog and PI3K/Akt, are deeply involved in cancer onset, progression, development of drug resistance and, specifically, cell stemness [148,149,150,151,152,153,154,155,156]. Altogether, these pathways form a complex network of molecular interactions that cooperate to govern the CSC traits regulating self-renewal, survival, cell fate decisions and differentiation.

#### 2.4.1. Wnt/β-Catenin Signaling

The canonical Wnt pathway, also known as the Wnt/β-catenin pathway, is triggered by the binding of the Wnt proteins to their specific membrane receptors in an autocrine or paracrine way. When Wnt ligands are absent, intracellular β-catenin is first phosphorylated by the intracellular β-catenin destruction complex and subsequently ubiquitinated by β-TrCP to be finally degraded at the proteasomal level. On the other hand, when Wnt proteins are present, they bind to specific membrane receptor complexes (Frizzled and LRP5/6 coreceptors), thus protecting β-catenin from degradation and allowing its translocation, facilitated by the APC/Axin/GSK-3 complex, into the nucleus where it binds to T cell factor/lymphatic enhancer factor (TCF/LEF) to promote the transcription of target genes, such as genes involved in cancer cell proliferation, survival, migration and stemness [157,158,159,160,161,162].

Wnt/β-catenin signaling has been found to play a significant role in regulating melanoma initiation and metastasis formation [163] and mediating the prometastatic activity of the CD133 stemness marker [164]. Recent studies pointed out that this intracellular pathway is also involved in the regulation of the biological and functional traits of melanoma CSCs. Wei and coworkers reported that, in melanoma cells, a downregulation of the ubiquitin–protein enzyme RNF128 correlates with EMT and the acquisition of stemness features through the activation of the Wnt pathway [165]. In line with these data, overexpression of the nucleolar protein NOP14 was found to suppress the biological functions and stemness traits of melanoma stem-like cells through the inactivation of the Wnt/β-catenin signaling [166]. Based on these observations, it has been proposed that targeting the Wnt/β-catenin pathway might represent a promising strategy for the effective eradication of CSCs and, therefore, for an effective treatment of melanoma patients [167,168,169].

#### 2.4.2. Notch Signaling

The Notch signaling pathway plays a crucial role in cell fate determination during embryogenesis; it is also deeply involved in developing, progressing and maintaining CSCs in different tumors [148,155,170,171]. Two groups of transmembrane proteins contribute to the activity of this pathway: Notch receptors, Notch1-4, and Notch ligands, including Jagged (JAG) 1 and 2 and Delta-like (DLL) 1, 2, 3 and 4. Upon the binding of a membrane-bound Notch ligand to a Notch receptor on the surface of a neighboring cell, the receptor undergoes two cleavages: the first occurs in the extracellular domain and is mediated by the ADAM family of proteases, while the second is performed at the level of the intracellular domain by γ-secretase and leads to the release of the Notch intracellular domain (NICD) into the cytoplasm. NICD then translocates into the nucleus to promote the transcription of target genes, such as Hes and c-myc, involved in the mechanisms of tumor progression [148,172,173,174,175,176]. However, the potential role of the Notch pathway in CSCs as a tumor promoter or a tumor suppressor signal is still controversial and seems to be related to the cellular setting [177,178].

Lin and coworkers reported that Notch4 is highly expressed in melanoma CSCs and promotes their metastatic and invasive ability [179]. Moreover, it has been reported that Notch1 increases the expression of the CD133 stemness marker in melanoma cells through the activation of the MAPK pathway.

CD133 controls VEGF and matrix metalloproteinase (MMP) expression, thus promoting tumor growth and angiogenesis [180]. Notch1 was also recently shown to mediate the proangiogenic activity of ALDH1A1-overexpressing melanoma cells [181]. In line with these observations, it has been demonstrated that Notch3 plays a key role in the maintenance of melanoma CSC plasticity; knockdown of Notch3 leads to a downregulation of the stemness markers CD133 and CD271, a depletion of the melanoma CSC subpopulation and an attenuation of the proangiogenic activity [182].

#### 2.4.3. Hedgehog Signaling

The Hedgehog (HH) signaling pathway is widely known to be deeply involved in embryogenesis, tissue homeostasis and regeneration; however, it has also been reported to be dysregulated in different types of cancers, as well as in melanoma [183,184,185]. The major components of this pathway include secreted HH proteins (Sonic, Indina, Desert), their cognate transmembrane receptor Patched (Ptch), the transmembrane protein Smoothened (Smo) and the three effector proteins Gli1-3. In the absence of the HH ligands, Ptch constitutively inhibits the activity of Smo; on the other hand, upon ligand binding, this inhibition is released and activated Smo triggers the nuclear translocation of the transcription factors Gli1-3, which in turn regulate the transcription of their target genes. Specifically, Gli1 and Gli2 are endowed with transcriptional activity while Gli3 mainly acts as a transcriptional repressor in the absence of ligands [186].

The role of HH signaling in the regulation of CSC traits and functions has been evidenced in different types of tumors [148,154,187,188]. Knockdown of Gli1 and Gli2 was found to restore drug sensitivity in vemurafenib-resistant melanoma cells [189]. Santini and coworkers observed that the HH/Gli pathway drives self-renewal and tumor initiation of ALDH+ melanoma CSCs. Moreover, in primary melanoma cells, the activation of this signaling cascade promotes the expression of Sox2, the transcription factor well known for its crucial role in self-renewal and tumorigenicity of melanoma CSCs [140,190]. In line with this observation, Horak et al. recently demonstrated that, in melanoma cells, activation of the HH pathway and Gli proteins directly promotes the expression of Slug, a hallmark factor of EMT as well as of cancer cell reprogramming into a stem-like phenotype [191].

#### 2.4.4. PI3K/Akt Signaling

Dysregulation of the phosphatidylinositol 3-kinase (PI3K)/Akt signaling cascade has been widely observed in various human solid cancers, such as melanoma, and found to correlate with disease development and progression [192,193,194,195,196,197,198].

This pathway is a multistep process that is triggered by the binding of different extracellular ligands to transmembrane tyrosine kinase receptors, leading to the phosphorylation of the membrane-associated kinase PI3K. Once activated, PI3K catalyzes the conversion of the membrane lipid phosphatidylinositol (3,4)-bis-phosphate (PIP2) into PIP3, which, in turn, promotes the recruitment of the serine and threonine kinase Akt to the cell membrane and its subsequent phosphorylation by various kinases. Ultimately, Akt activates the mammalian target of rapamycin mTOR (specifically the mTORC1 complex), regulating the downstream effectors, which are deeply involved in cancer cell proliferation, survival, angiogenesis and drug resistance [199,200,201,202,203].

Accumulating evidence supports that the PI3K/Akt pathway is overactivated and plays a relevant functional role in CSCs [148,204]. In BRAFV600E+ melanoma cells, an aberrant activation of this intracellular pathway has been reported to mediate resistance to BRAF and MEK inhibitors and to support the acquisition of stem-like properties [205,206]. Corrales and coworkers demonstrated that, in melanoma cells, a decrease in sensitivity to MAPK/ERK inhibitors is accompanied by higher activity of the PI3K/Akt pathway, which is particularly evident in mesenchymal-like cells supporting their acquisition of stem-like features [207]. In line with these observations, it was reported that a CD133-dependent activation of PI3K/Akt/mTOR signaling is required for the maintenance of stemness traits and drug resistance in melanoma cells [64,87]. Vasculogenic mimicry (VM), the potential of cancer cells to differentiate into endothelial-like cells directly contributing to the “angiogenic switch” and promoting angiogenesis, was first observed in melanoma CSCs [180,208,209]. Notably, it has been reported that the PI3K/Akt pathway plays a pivotal role in the VM ability of melanoma CSCs [210,211].

Together, these observations strongly support that targeting the PI3K/Akt/mTOR pathway could represent a successful strategy to overcome therapy resistance while specifically suppressing CSCs and tumor angiogenesis in melanoma.

## 3. Cell-to-Cell Communication between Melanoma CSCs and the Tumor Microenvironment

A growing body of evidence strongly supports that a deep interplay exists between cancer cells and their surrounding TME. The TME encompasses different types of cells, such as immune cells, CAFs, endothelial cells, keratinocytes and adipocytes; other factors present in the tumor niche include extracellular matrix components, hypoxia and signaling molecules. Cancer cells, particularly melanoma cells, release molecular messages to surrounding cells to promote a tumor-sustaining microenvironment. On the other hand, the cancer microenvironment promotes tumor growth, progression, invasiveness, angiogenesis and drug resistance [69,212,213,214,215,216]. The molecular messages involved in this bidirectional communication involve growth factors, chemokines and cytokines, as well as EVs carrying a specific molecular cargo of mRNAs, miRNAs, DNA, proteins and lipids. Cells secrete EVs into the extracellular space to release their molecular cargo to near and far cells, thus affecting their biological features [217,218,219,220,221,222]. Notably, a complicated crosstalk also exists between CSCs and their surrounding cells and plays a pivotal role in CSC self-renewal, maintenance and resistance to therapies [69,70,71,223,224,225]. Herein, we specifically address the interplay between melanoma CSCs and their surrounding cells in the TME and its role as a potential target in melanoma therapies.

### 3.1. Melanoma CSC–Immune Cell Interplay

**Figure 2:**
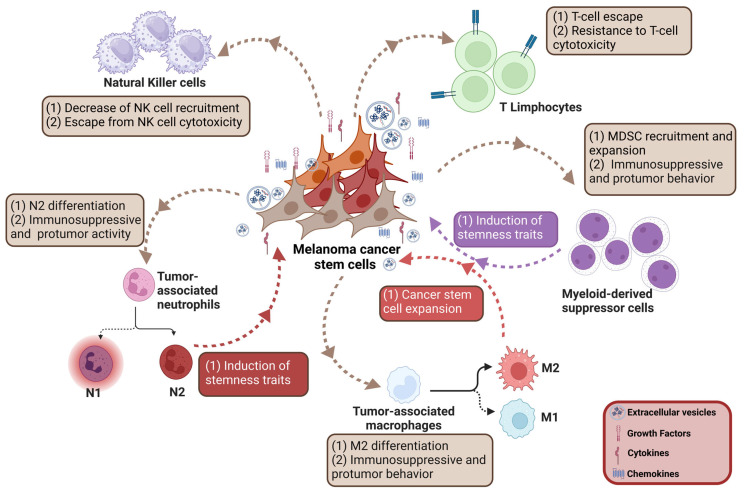
Schematic overview of the cell-to-cell bidirectional communication between melanoma CSCs and immune cells within the TME. Melanoma CSCs decrease immune cell recruitment while escaping their cytotoxicity (T cells, NK cells), promote the conversion of TANs and TAMs cells into a protumoral phenotype (N2 and M2, respectively) and recruit MDSCs immunosuppressive cells. Conversely, immune cells promote the induction of stemness traits and cell expansion in CSCs. TME, tumor microenvironment; MDSCs, myeloid-derived suppressor cells.

Based on the remarkable improvements in immunotherapy for cutaneous melanoma patients, the interplay between melanoma cells and infiltrated immune cells has recently gained increasing attention [49,212,214,226,227,228,229]. As discussed above, primary and acquired resistance frequently occurs in patients challenged with immune checkpoint inhibitor therapies and accumulating evidence has recently highlighted the involvement of CSCs in this undesired event. Specifically, melanoma CSCs have been widely shown to possess a striking ability to escape host immune surveillance (immune evasion) and, concurrently, to eliminate antitumor immune cells or to selectively recruit immune cells endowed with peculiar immunosuppressive and tumor-promoting features [63,230,231]. A strict correlation has been observed between melanoma CSCs and tumor-infiltrating immune cells [232,233] (Figure 2).

#### 3.1.1. Interactions with Adaptive Immune Cells

T lymphocytes are the main component of the adaptive immune system, characterized by the ability to gain functional/effector abilities according to the immunological context.

The key role of tumor-associated antigens (TAAs) in eliciting melanoma antitumor immunity is well recognized. These TAAs include melanoma antigen recognized by T cells (MART-1), gp100, tyrosinase, tyrosine-related protein 1/2 (TRP1/2) but also cancer–testis antigens, such as NY-ESO1, MAGE-A3 and MAGE-A4. The immunogenicity of these antigens is supported by the observation that their cytotoxic T cell-mediated targeting results in a significant decrease in tumor burden in mice and melanoma patients [234,235,236]. However, an inadequate adaptive immune system response is frequent in melanoma, suggesting the presence of a subpopulation of dedifferentiated tumor cells similar to CSCs characterized by a low immunogenic profile and an immunosuppressive behavior deeply associated with tumor growth and progression [226,230]. Consistently, it has been reported that ABCB5+ and CD271+ melanoma cells express very low levels of the differentiation marker MART-1 as well as of cancer–testis antigens [92,237,238]. Landsberg and coworkers demonstrated that immunotherapy-resistant melanoma cells xenografted in nude mice models undergo a dedifferentiation process characterized by the concurrent gain of the stemness marker nerve growth factor receptor (NGFR) and loss of TAAs [239]. Similar results were also reported in in vitro and in clinical studies [240,241]. Interestingly, Huang et al. reported that inhibiting the phosphorylation (i.e., activation) of eukaryotic translation initiation factor 4E (eIF4E) in a PTEN KO transgenic melanoma mouse model prevents tumor cell dedifferentiation, resulting in increased expression of TAAs (MART-1 and gp100), thus facilitating their recognition and eradication by cytotoxic T cells. Moreover, phopsho-eIF4E levels were found to correlate negatively with MART-1 expression in melanoma biopsies [242]. Taken together, these data collectively support that differentiated melanoma cells expressing TAAs can be specifically eradicated by cytotoxic T cells; on the other hand, the loss of expression of these antigens in dedifferentiated CSCs is strictly associated with the activation of immune escape. However, it must be underlined that some contradictory results have been reported [239,243] and they have been explained by the possible involvement of different types of TAAs in the T cell-induced eradication of melanoma CSCs [228,244].

It is now well accepted that, in spite of a correct expression of immunogenic antigens, the immune evasion ability of CSCs can be associated with defective antigen presentation or processing. In addition to the reported decreased expression of TAA, it has been recently shown that melanoma CSCs express low levels of molecules involved in antigen presentation, such as major T cell histocompatibility (MHC) class I proteins [237,238]. So, even if TAAs are normally expressed, the absence of functional costimulatory MHC class I proteins hinders cytotoxic T cell activation, thus contributing to immune evasion. Similarly, an impairment of T cell activity can be triggered by the presence of a negative costimulation. For instance, negative modulators of T cell functions, such as PD-L1, PD-1 and CD86, have been reported to be highly expressed in melanoma CSCs, mediating their immune escape behavior. Thus, the low immunogenic features of melanoma CSCs are strictly correlated to a repression of differentiation antigens and costimulatory antigen presentation molecules as well as to an overactivation of negative costimulatory machinery.

In recent years, accumulated evidence has revealed that different melanoma CSC markers are also directly involved in the mechanisms of tumor cell resistance to T cell cytotoxicity. Tripartite motif-containing 28 (TRIM28) is a well-known marker of cancer cell stemness being deeply involved in the maintenance of stem cell renewal [245]. Czerwinska and coworkers reported that melanoma tumors expressing high levels of TRIM28 are significantly depleted with infiltrating cytotoxic T cells, as well as B cells and helper T cells, and suggest that this molecule might be considered as a good predictor of a “stemness high/immune low” melanoma phenotype [246]. It has been shown that melanoma cells resistant to MART-1 T cells express high levels of the stemness marker NGFR. Knockdown of this receptor sensitizes melanoma cells to MART-1 T cells, supporting its specific role in melanoma CSC resistance to T cell cytotoxic activity [241]. Kim and coworkers reported that Nanog triggers the upregulation of the tubule-associated protein 1 light chain 3 beta (LC3), which, in turn, activates the EGFR/Akt signaling, thus rendering melanoma CSCs resistant to T cell recruitment and cytotoxicity [146]. Sox2, another very well-known marker of melanoma stemness, has also been described as a player in the mechanisms of immune resistance. Specifically, Wu et al. recently demonstrated that Sox2 is able to promote the expression of immunosuppressive genes, such as PD-L1 and IDO1, favoring melanoma CSC-mediated depletion of T cell cytotoxic activity; in line with this observation, epigenetic suppression of Sox2 was observed to synergize with anti-PD-1-based immunotherapy in melanoma mouse models [247]. Finally, T cell inhibition may be directly triggered by tumor cell-secreted cytokines, including IL-4, IL-10 and TGF-β; it has been reported that ABCB5+ melanoma cells express and release in the TME high levels of TGF-β-related factors [84].

#### 3.1.2. Interactions with Innate Immune Cells

Natural Killer (NK) cells, a subpopulation of large granular lymphocytes, are effector lymphocytes of the innate immune system that provide host defense against tumors. They directly interact with tumor cells, as well as CSCs, or indirectly with other cells to regulate tumor growth within the TME [248,249]. Different mechanisms have been observed to be activated in melanoma CSCs to escape from cytotoxicity elicited by NK cells. Melanoma cells undergoing EMT were shown to express high levels of the NK-protective HLA-I protein on their surface and to downregulate the expression of various NK cell-activating receptors [250]. The integrin beta-like protein 1 (ITGBL1) is a factor highly expressed in melanoma CSCs; secreted ITGBL1 protein was found to impair NK cell cytotoxicity [251]. It is well known that the β-adrenergic signaling pathway is endowed with a protumor activity through the β3-adrenoreceptor (β3-AR) and blocking β3-AR activation protects against the development of different types of tumors. Calvani and coworkers reported that the pharmacological inhibition of β3-AR reduces the expression of stemness markers in melanoma cells and this effect is associated with an increased recruitment of NK cells. This evidence supports a key role of this receptor as a stemness marker in melanoma cells being deeply involved in tumor immune escape mechanisms [252]. More recently, Lehmann et al. reported that the immune surveillance exerted by NK cells is bypassed by melanoma cells expressing high levels of the NGFR stemness marker. Specifically, by using in vitro and in vivo experimental models, these authors could observe that NGFR protects tumor cells from NK cell-mediated clearance by downregulating NK cell-activating ligands and lipid reprogramming [253]. In cutaneous melanoma, infiltrated NK cells are functionally impaired. A dysregulation of the activating NKG2D receptor expressed in NK cells and involved in tumor recognition has been highlighted as a factor underlying their defective behavior. This receptor signaling is crucial in the regulation of NK cell cytotoxic activity and cytokine production. It has been demonstrated that very low levels of NKG2D can be detected in NK cells from melanoma tissues enriched with the stem cell marker CD133+ [254].

Immunosuppressive cells, such as tumor-associated neutrophils (TANs), tumor-associated macrophages (TAMs), myeloid-derived suppressor cells (MDSCs), DCs and adoptive immune cells like the regulatory T cells (Tregs), are the cellular components of the TME hindering the protective immunosurveillance of neoplasia, impairing the antitumor immune responses and promoting tumor growth and progression [255].

TANs are a type of white blood cells, representing the most abundant immune cells in the TME. They are involved in the early phases of inflammation and represent the first line of defense of the innate immune response, being endowed with an immunostimulatory effect. However, cancer cells release different types of cytokines, such as TGF-β, IL-6 and IL-8, that promote TAN polarization from an antitumor (N1) to an immunosuppressive and protumor phenotype (N2) [256,257]. Specifically, N1 cells are characterized by slow proliferation and by the release of high levels of tumor necrosis factor-alpha (TNF-α). In contrast, N2 cells are endowed with immunosuppressive and protumor activities favoring the release of NETs (neutrophil extracellular traps, a network of extracellular strings of DNA), ROS (reactive oxygen species), MMP-9, IL-8 and IL-6 [257,258]. Recently, Modestino and colleagues reported that melanoma cells induce chemotaxis as well as the activation and release of NETs from polymorphonuclear neutrophils; moreover, high levels of MMP-9, IL-8, NET, granulocytes and monocyte colony-stimulating factors were reported in patients with advanced melanoma when compared to healthy controls [259]. In line with these data, a high presence of TANs was observed in melanoma patients and found to correlate with a low therapeutic outcome [260]. Interestingly, we recently demonstrated that melanoma CSCs release soluble factors, such as TGF-β, IL-6 and IL-8, able to promote neutrophil recruitment and their switch toward the N2 phenotype, mediated by the activation of the ERK, p38 and STAT3 signaling pathway and the concurrent overexpression of CXCR2 and NF-κB. Moreover, the conditioned medium from melanoma CSCs promoted ROS production, cytokines and MMP-9 secretion and NET release from neutrophils. Notably, we could also observe that CSC-activated neutrophils could endow melanoma cells with peculiar stemness traits (i.e., ABCG2 expression and melanosphere formation) [261]. These results strongly support the existence of a bidirectional interplay between CSCs and neutrophils in melanoma.

Macrophages are cells of the innate immune system that derive from the differentiation of monocytes in tissues. Like neutrophils, two opposite TAM polarization states have been described, named M1 and M2 [262]. The former is characterized by the production of proinflammatory cytokines such as TNF-α, IL-1β, IL-6 and IL-12 and promotes an immune response against the tumor. The latter secrete anti-inflammatory cytokines, such as IL-10, CCL18 and TGF-β, and favors an immunosuppressive environment supporting tumor growth and progression [263,264]. M2 cells were shown to promote melanoma cell proliferation, invasion, metastasis and angiogenesis and to be deeply involved in the development of drug resistance [265,266,267,268,269]. Moreover, in different types of cancers, TAMs have been reported to be recruited and educated toward the M2 phenotype by the CSC subpopulations through the release of chemokines and interleukins; on the other hand, M2 cells establish a TME niche favorable for CSC survival and maintenance of stemness properties [270,271]. These data support a strict bidirectional crosstalk between CSCs and macrophages in the TME. Glucosylceramide synthase (GCS) is an enzyme well known to play an important role in sphingolipid biosynthesis, which is necessary for the maintenance of melanoma CSCs. Ghosh and coworkers demonstrated that tumor hypoxia induces GCS expression in melanoma cells, correlated with a significant increase in the melanoma CSC subpopulation. Mechanistically, hypoxia induces the release of TGF-β from TAMs and Tregs (the specialized subpopulation of T cells that act to suppress immune response), which, in turn, promotes GCS expression in melanoma cells, thus favoring the expansion of CSCs. Moreover, siRNA-based silencing of GCS abrogates the immunosuppressive ability of TAMs and Tregs. Together, these data support the notion that a bidirectional communication exists between melanoma CSCs and tumor-infiltrating immune cells and plays a pivotal role in CSC maintenance and tumorigenicity [272].

MDSCs are a heterogeneous group of myeloid cells derived from hematopoietic cells residing in the bone marrow and characterized by a peculiar immunosuppressive activity [273]. Specifically, they are endowed with the ability to impair both adaptive and innate immune responses in different pathological conditions, including cancer, facilitating immune evasion [274,275,276,277,278,279,280,281]. Moreover, they play a crucial role in promoting EMT and boosting CSC stemness. On the other hand, CSCs attract MDSCs, favoring their infiltration, expansion and immunosuppressive abilities [282]. In breast cancer, MDSCs have been shown to play an important role in escaping the immune response as well as in promoting the tumorigenic and metastatic capacity and the stemness features of cancer cells through the activation of the STAT/Notch signaling pathway [283,284]. MDSCs were also reported to promote immune evasion in melanoma cells by inhibiting T cell activity and macrophage-mediated phagocytosis [227,285,286] and to confer stemness properties to tumor cells by activating the IL-6/STAT3 signaling pathway [287]. Notably, in preclinical studies, Shidal and colleagues demonstrated that CD133+ melanoma CSCs in the B16F10 melanoma murine model are associated with the recruitment of high amounts of immunosuppressive cells, such as MDSCs but also M2 macrophages and Treg cells, during tumor formation. Mechanistically, these authors showed that a low expression of miR-92 in melanoma CSCs is associated with the activation of the integrin α/TGF-β axis to promote intratumoral immunosuppression and enhance tumorigenesis [288].

### 3.2. Melanoma CSC–Endothelial Cell Interplay

The process of angiogenesis, the generation of new capillary blood vessels from pre-existing ones, is now considered a hallmark of cancers, including melanoma [289,290]. New vessel formation is necessary to provide oxygen and critical nutrients for tumor growth and facilitate metastatic dissemination [291]. Several growth factors signaling pathways are involved in angiogenesis. In particular, the vascular endothelial growth factor (VEGF) family (VEGF-A, VEGFB, VEGF-C, VEGF-D, PlGF) and their receptors, VEGFR-1, VEGFR-2 and VEGFR-3, have been consistently reported to be present in melanoma tissues. Activation of this VEGF/VEGFR axis, through the engagement of different intracellular pathways (i.e., MAPK/ERK, PI3K/ERK, PKC and FAK), facilitates melanoma cell proliferation, migration, survival and permeability [292].

It is now well recognized that melanoma CSCs, particularly those located at the tumor margins, express and release several factors deeply involved in the angiogenesis process in their surrounding microenvironment. By setting up a tri-layered melanoma model composed of CSCs, fibroblasts, mesenchymal stem cells and endothelial cells, Lopez de Andrés and coworkers recently observed that embedded cells show high proliferation accompanied by an early onset of vascularization [293]. ABCG2+ and CD133+ melanoma cells were reported to secrete proangiogenic factors such as VEGF and its receptor VEGFR-2, Tie2, angiopoietin and MMP-2/-9 [82,180]. In melanoma cells expressing high levels of CD271, integrin α5β1, through its downstream ERK pathway, regulates the proangiogenic secretome, promoting vessels formation [294]. Similarly, ALDH1A1 overexpression in melanoma cells was found to correlate with an upregulated secretion of proangiogenic factors promoting angiogenic traits in surrounding endothelial cells through the activation of the intracellular Notch signaling cascade [181]. The NOP14 nucleolar protein is known to be involved in the regulation of melanoma pathogenesis. Li and colleagues reported that NOP14 suppresses the stemness and functions of melanoma CSCs through the inactivation of the Wnt/β-catenin intracellular signaling; specifically, its overexpression significantly impairs proliferation, migration and the proangiogenic potential of these cells [166].

**Figure 3:**
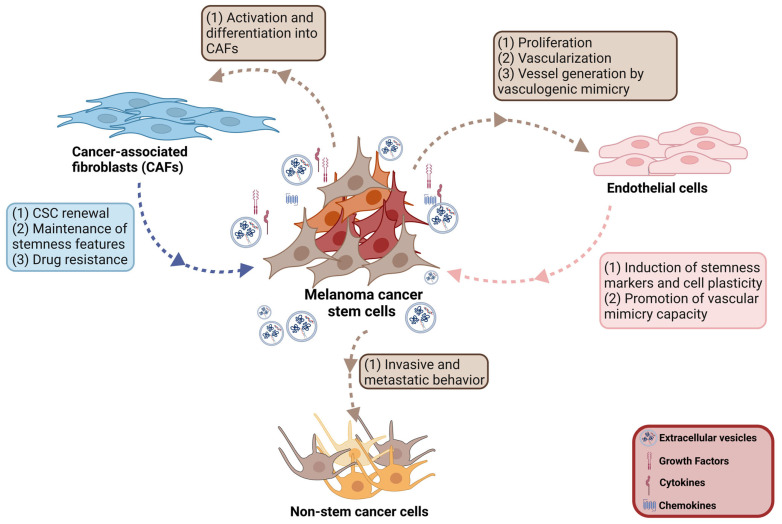
Schematic overview of the cell-to-cell bidirectional communication between melanoma CSCs and stromal cells (endothelial cells, CAFs) within the TME, and between melanoma CSCs and non-stem cancer cells within the tumor bulk. Melanoma CSCs trigger the activation of stromal fibroblasts into CAFs and promote neoangiogenesis by inducing endothelial cell proliferation and new blood vessel formation and acquiring an endothelial phenotype (vasculogenic mimicry, VM). Conversely, CAFs and endothelial cells promote the expression and maintenance of stemness traits in melanoma CSCs. Melanoma CSCs endow non-stem cancer cells in the tumor bulk population with high metastatic properties. CAFs, cancer-associated fibroblasts; TME, tumor microenvironment.

VM, the ability of tumor cells to acquire endothelial cell morphology and function, is a process that was first reported to occur in melanoma [295]. In particular, accumulating evidence indicates that melanoma CSCs, characterized by a high level of plasticity, are able to develop an endothelial-like phenotype for the generation of new tumor-derived vessels, giving rise to an alternative microcirculation that is independent of endothelial cells [295,296,297]. The PI3K/Akt signaling pathway has been shown to be deeply involved in this “angiogenic switch” that allows tumor dissemination and metastasis [211]. Moreover, vascular endothelial cadherin (VE-cadherin), a cell–cell adhesion protein commonly expressed by endothelial cells, has been found to be overexpressed in different types of tumors where it contributes to disease progression/aggressiveness and to the acquisition of the VM phenotype [298]. ABCB5+ melanoma cells were reported to express endothelial/proangiogenic molecules as well as specific VM markers, such as VEGF and its receptors, Tie2 and VE-cadherin [299]. Moreover, CD133+ and ABCG2+ melanoma stem cells were shown to overexpress proangiogenic factors and to be characterized by tube-formation capacity [82,180]. Consistently with these observations, CD271+ melanoma stem cells were found to be endowed with VM through activation of the VEGFR/PKC pathway [209]. Syndecan-1, a cell surface heparan sulfate proteoglycan, has been shown to play an important role in the VM process of melanoma CSCs [300]; moreover, Shih et al. recently demonstrated that the heparan sulfate binding angiopoietin-like 4 (ANGPTL4) protein, highly expressed in melanoma CSCs, is deeply involved in the tube-forming ability of these cells in vitro [301]. These data were further confirmed by in vivo studies demonstrating that CD133+ melanoma cells xenografted into nude mice promote tumor growth associated with a significant angiogenesis process [302]. Interestingly, using a two-dimensional (2D) melanoma–endothelium co-culture model, Hsu and colleagues could demonstrate that endothelial cells, through the activation of the Notch3 signaling, facilitate melanoma CSC plasticity by inducing the expression of stemness markers, and promote their VM capacity [182]. Together, these data support that a strict two-way communication between CSCs and endothelial cells is present in melanoma TME and plays a crucial role in regulating the angiogenic process (Figure 3).

### 3.3. Melanoma CSC-CAF Interplay

CAFs are an important component of the tumor niche, being deeply involved in the promotion of tumor growth and progression, suppressing antitumor immunity and acquiring resistance to cancer therapies [303,304,305,306]. Specifically, a close interaction has been shown between tumor cells and neighboring CAFs in melanoma. CCN2 is a protein belonging to the cellular communication network (CCN) family; high levels of CCN2 have been observed in normal tissue repair and pathologies, including cancers, while it is not expressed by dermal fibroblasts. In human melanoma biopsies, CCN2 expression has been found to negatively correlate with patient survival. Tsang and coworkers reported that tumor cells recruit dermal fibroblasts, fostering their expression of the stem cell marker Sox2 and activation into a CAF phenotype in a CCN2-dependent manner [307]. Eph receptors are the largest family of RTKs and are different from other RTKs by binding to cell-bound ligands (ephrins), being deeply involved in cell–cell junctions and in the regulation of cell adhesion and survival. In particular, the EphA3 isoform has been shown to be overexpressed in melanoma-associated CAFs, while its downregulation is associated with the inhibition of tumor growth and progression [308]. miR-214 expression has been found to be significantly upregulated in malignant melanomas and to act as a prometastatic miRNA through the modulation of a complex molecular pathway including transcription factors, adhesion molecules and the anti-metastatic miR-148b [309]. Orso and colleagues reported that miR-214 is highly expressed in CAFs located in melanoma tissues. Treatment of cancer cells with conditioned medium or EVs derived from miR-214-enriched CAFs promotes melanoma cell migratory and invasive behavior. Interestingly, melanoma cells are able to induce miR-214 production in stromal CAFs, which is then released via EVs and subsequently uptaken by cancer cells. These data support that a bidirectional interplay exists between melanoma cells and CAFs in the TME and plays an important role in coordinating tumor metastatic dissemination [310].

It is now well accepted that tumor-associated CAFs are also deeply involved in CSC renewal, maintenance of stemness traits and drug resistance [311,312]. By means of in vitro and in vivo studies, Kinugasa et al. demonstrated that CAFs expressing the stemness marker CD44 sustain the stemness features of melanoma CSCs via a direct interaction and promote cancer cell drug resistance [313]. More recently, it has been reported that the intracellular Notch1 signaling pathway in CAFs dictates the plasticity of human metastatic melanoma cells. Specifically, Notch1-overexpressing CAFs were found to induce a sustained suppression of melanoma cell stemness; conversely, Notch1-silenced CAFs significantly increased the stemness properties of melanoma CSCs by upregulating the expression of the stemness markers Sox2, Oct4 and Nanog [314] (Figure 3).

Overall, melanoma CSCs and neighboring CAFs in the tumor stroma display a pathological interplay; however, further studies are needed to elucidate the underlying molecular and cellular mechanisms.

## 4. Cell-to-Cell Communication between CSCs and Non-CSCs in Melanoma

Tumors are generally characterized by a high degree of heterogeneity and plasticity containing cells with different malignant potentials; accumulating evidence supports that the communication between these cells plays an important role in tumor progression and drug resistance [315,316,317,318]. In different tumors, it has been shown that CSCs can endow bystander cells of the bulk population with self-renewal and stemness properties through the horizontal transfer of specific molecular signals [319,320,321,322]. In melanoma, highly metastatic CSCs release exosomes carrying high levels of specific miRNAs, including miR-1268a and miR-4535. Once internalized by target melanoma cells, CSC-derived exosomes deliver both miR-1268a and miR-4535, which in turn foster the invasive behavior and metastatic colonization capability of these cells through the inhibition of the autophagic pathway [323,324]. In line with these observations, it has been demonstrated that EVs derived from melanoma CSCs package and deliver miR-592 to differentiated melanoma cells. Uptaken miR-592 inhibits the expression of its target gene protein tyrosine phosphatase non-receptor type 7 (PTPN7), leading to the activation of the MAPK/ERK signaling pathway and, ultimately, to the promotion of melanoma cell metastatic ability [325]. Interestingly, long non-coding RNAs (lncRNAs) have also been found to be frequently carried by exosomes mediating the communication between the different cell subpopulations of the tumor mass. Chen and coworkers recently reported that the lncRNA Gm33149 is encapsulated into exosomes originating from melanoma CSCs and is then transferred to parental melanoma cells. Mechanistically, upon being uptaken by non-CSCs, Gm33149 competitively binds to miR-5623-3p, thereby activating the Wnt pathway and fostering their acquisition of a high metastatic behavior [326]. So far, studies addressing the horizontal transfer of stemness traits from CSCs to non-stem cells within the melanoma mass are still missing.

Overall, these findings support that exosomal molecular cargos are deeply implicated in the horizontal crosstalk between the different melanoma cell subtypes, being responsible for the transfer of tumor aggressiveness. These cargos not only represent potential biomarkers of tumor progression, but may also provide valuable support for the development of novel therapeutic strategies leading to an improvement in melanoma patient outcome (Figure 3).

## 5. Targeting CSCs in Melanoma

Chemoresistance to conventional anticancer treatments and immune evasion properties are widely recognized as distinctive properties of CSCs. Thus, targeting the mechanisms operating in CSCs, in combination with standard treatments aimed at eradicating the bulk tumor cell populations, might open new avenues for cancer treatment strategies [327,328,329]. Notably, therapeutic approaches directed against surface and intratumoral targets, such as markers of drug resistance and intracellular pathways in the context of melanoma CSCs, have been recently explored (Table 1 and Table 2).

CD271 is a key molecular marker of melanoma CSCs, and its targeting might represent an effective therapeutic strategy for melanoma patients. Morita and coworkers developed a humanized anti-CD271 monoclonal antibody (mAb) and found that it exerts a cytotoxic activity against CD271+ cell lines. Moreover, in vivo studies showed that this antibody significantly inhibits tumor growth in human melanoma cell xenograft models by depleting the CD271+ cell subpopulation [101]. In line with these observations, it has been shown that mAb-mediated blockade of CD47, a well-known marker of melanoma metastasis and immune evasion, coupled with a CD271 cytotoxic antibody linked to the toxin saporin (CD271S, anti-CD271 mAb-saporin), significantly impairs tumor metastatic behavior in melanoma patient-derived xenografts [94]. In contrast, Saltari et al. reported that activation of the CD271 intracellular domain by a short β-amyloid-derived peptide (Aβ(25-35)), in combination with chemotherapy or targeted therapy, significantly decreases melanoma metastasis in a zebrafish xenograft model as well as tumor growth in mice [330]. Thus, further studies are needed to confirm the reliability of CD271 as an effective target to overcome melanoma progression and drug resistance. In this context, the relevant roles of CD271 in the central nervous system must be considered; appropriate delivery methods need to be developed to specifically target CD271 expressed in the CSC population.

Therapeutic approaches aimed at targeting the CSC marker CD133 have been developed and explored through in vitro and in vivo studies. Rappa and colleagues demonstrated that monoclonal antibodies directed against two distinct epitopes of this marker exert a significant and dose-dependent antitumor activity in melanoma cells. Moreover, the downregulation of CD133, by means of short hairpin (sh) RNAs, markedly reduces in vitro and in vivo the metastatic ability of these cells [331]. Photochemical internalization (PCI) is a laser-controlled technology utilized to promote endosomal escape and specific intracellular delivery of hydrophilic anticancer drugs. AC133-saporin is an immunotoxin complex consisting of a CD133 mAb linked to saporin (AC133-saporin). Bostad and coworkers took advantage of the PCI technology by employing a photosensitizer that induces endosomal disruption and subsequent endosomal escape of internalized anticancer drugs, to investigate the potential of AC133-saporin for targeting the CD133+ population of melanoma cells. These authors observed that treating melanoma cells with PCI/AC133-saporin significantly reduces tumor growth in vitro and in vivo, suggesting this approach as a novel rational strategy for eradicating CD133+ melanoma CSCs [332].

Therapeutic approaches have also been developed to specifically target the melanoma CSC marker CD20. In a chemotherapy-refractory melanoma patient with metastatic disease, intra-lesional injections of the monoclonal anti-CD20 antibody rituximab, together with dacarbazine treatment, induced regression of treated metastasis. Notably, rituximab treatment decreased the number of CD20+ positive cells in metastatic lesions and no treatment-related toxicity could be observed [333]. Winkler and coworkers reported results from a clinical study carried out in seven metastatic melanoma patients receiving rituximab injections for 1 year. All patients had previously experienced different antitumor approaches. Treatment was found to be well tolerated although two patients experienced infusion reactions (grade 2) during the first infusion, but there were no more serious side effects. Five patients showed stable disease, while two patients had progressive disease. Median progression-free survival was 6.3 months, median overall survival was 14.7 months and one patient was still alive 18 months after the start of treatment. These authors suggested that the beneficial toxicity profile of rituximab supports that its combination with other standard systemic treatments (i.e., PD-1 antibodies) might increase therapeutic efficacy and should be further investigated in clinical studies involving large patient cohorts [334]. In line with this suggestion, it has been demonstrated that targeting CD20+ melanoma cells with rituximab significantly potentiates the BRAF inhibitor (vemurafenib)-mediated cell killing [335]. Recently, different therapeutic strategies involving CD20 antibodies were developed. In this context, immunoliposomes conjugated to CD20 antibodies and loaded with the standard anticancer compound vincristin (VCR-Lip-CD20) were reported to significantly decrease the ability of melanoma cells to form melanospheres in vitro and tumor growth in melanoma xenograft mice [336]. Aptamers are short synthetic single-stranded RNA/DNA oligonucleotides that specifically bind to different cell surface molecular targets such as proteins; they have different advantages over antibodies, being highly specific, non-immunogenic and relatively small in size, thus enabling a better drug penetration in tumor cells [337,338]. It was shown that salinomycin-loaded lipid–polymer nanoparticles bound to anti-CD20 aptamers (CD20-SA-NPs), upon being uptaken into CD20+ melanoma cells, exert an important cytotoxic activity against these cells while impairing their ability to form melanospheres [339]. In line with these data, Chen and colleagues reported that anti-CD20 aptamer-modified exosomes loaded with the standard chemotherapy drug adriamycin (ACEXO) efficiently accumulate in melanoma cells and reduce the number of melanospheres; importantly, these results were also confirmed in preclinical studies performed in melanoma tumor-bearing mice [340].

By means of in vitro and in vivo studies, it has been pointed out that lipid nanoparticles coated with hyaluronic acid (HA), a well-known ligand of CD44, and loaded with the taxane chemotherapy drug paclitaxel (PTX-loaded HA-SLNs) suppress the subpopulation of CD44+ melanoma cells [341]. Similarly, albumin nanoparticles functionalized with HA to specifically target CD44-overexpressing cells and filled with all-trans retinoic acid (HA-eNPs/ATRA) were found to suppress the tumorigenicity as well as the metastatic potential of CD44-enriched melanoma cells [342]. RG7356 is a recombinant anti-CD44 immunoglobulin G1 humanized monoclonal antibody. The efficacy of RG7356 has been investigated by Menke-van der Houven van Oordt and colleagues in a first-in-human phase I trial in patients with locally advanced or metastatic cancers expressing high levels of CD44, including melanoma. Patients unresponsive to previous treatments were intravenously treated with escalating doses of the compound. The treatment was well tolerated, showing only minor adverse effects. However, the clinical efficacy was low, with only 21% of patients experiencing a disease stabilization of a median of 12 weeks. Based on these results, these authors suggested that additional investigations of the efficacy of RG7356, possibly in combination regimens, should be carried out [343].

Specific compounds have also been developed to target drug resistance markers. In particular, monoclonal antibodies directed at ABCB5 were reported to reverse melanoma cell resistance to doxorubicin [84]; however, further investigations are warranted to confirm these data. In our laboratory, we demonstrated that the natural compound δ-tocotrienol decreases the ability of melanoma cells to form melanospheres and promotes the disaggregation of melanospheres, suppressing the expression of the ABCG2 marker [78].

The intracellular stemness marker ALDH is another potential target for novel cancer therapies; different inhibitors of multi-isoform ALDH (specifically ALDH1A1, ALDHA2, ALDH3A1) have been developed and their biological activity has been evaluated in different types of tumor cells, including melanoma cells [132]. Interestingly, it has been shown that ALDH1 can induce the activation of nifuroxazide (a member of the 5-nitrofurans family); of interest, activated nifuroxazide targets ALDH1+ melanoma cells, leading to the suppression of their stemness features [131]. A whole-cell melanoma vaccine genetically modified toward a CSC phenotype characterized by high levels of ALDH1 (AGI-101H) was applied in advanced melanoma patients, with resected and non-resected tumors, and combined with surgery in patients with recurring metastases. AGI-101H treatment was associated with a significant increased patient survival (11–19 years) and found to trigger the generation of cytotoxic CD8+ T cells specific for ALDH1A1 [344]. More recently, Liao and coworkers developed a dual ALDH1A1+ALDH1A3 peptide–dendritic cell vaccine and found that it induces suppression of tumor growth by eliciting a peculiar T cell immunity that specifically targets ALDH+ melanoma cells [133].

Last but not least, different natural and synthetic compounds, such as triphenylmetane gentian violet, lunasin, retinoic acid and chelerythrine chloride, have also been shown to specifically target the melanoma CSC subpopulation by regulating the expression/activity of the embryonic transcription factors Oct4, Sox2 and Nanog [345,346,347,348].

Inhibition of the intracellular signaling pathways involved in the promotion of cancer cell stemness is also considered a potential therapeutic approach for cancer patients.

Several anticancer compounds were found to exert their activity by targeting the Wnt/β-catenin pathway in CSCs and some of them are in clinical trials for the treatment of patients with Wnt-driven tumors [149]. Pimozide is an antipsychotic drug approved by the FDA in 2011. Accumulated evidence supports that this compound is endowed with peculiar cytotoxic properties on cancer cells, including melanoma cells, by targeting the CSC subpopulation [169,349]. Specifically, it has been reported that pimozide inhibits the signal transducer and activator of the transcription (STAT-3 and 5) pathway, which is overactivated in CSCs, thus significantly impairing CSC features. Interestingly, recently accumulated evidence supports that this compound is endowed with peculiar cytotoxic properties on cancer cells, including melanoma cells, by specifically targeting the CSC subpopulation [169]. A novel anti-CSCs drug (35b), derived from the natural chemical scaffold of symplostatin 4 (a natural anti-malaria depsipeptide), was reported to exhibit a significant inhibitory effect on melanoma growth by reducing CSC traits, such as melanosphere formation and ALDH expression, by impairing the Wnt/β-catenin signaling pathway [168]. Moreover, the natural compound morin (3,5,7,20,40-pentahydroxyflavone), a natural polyphenol isolated from members of the Moraceae family, has been reported to inhibit the growth of many types of cancer cells, including melanoma CSCs. Mechanistically, it has been demonstrated that morin significantly inhibits the growth as well as the stemness features (self-renewal and sphere-formation capacity) of melanoma cells overexpressing CD133, by increasing the expression of miR-216a while hampering its downstream target Wnt3 [350].

Targeting the Notch signaling pathway has been proposed as an effective strategy to eradicate the CSC subpopulation in different tumors [351,352]. The γ-secretase inhibitors RO4929097 and honokiol were reported to reduce the proliferation and the ability to acquire stemness features of melanoma cells [353,354]. However, Keyghobadi et al. demonstrated that only short-term inhibition of the γ-secretase/NICD axis, using the γ-secretase inhibitor DAPT (N-[N-(3,5-difluorophenacetyl)-L-alanyl]-Sphenylglycine t-butyl ester), is associated with reduced proliferation and stemness traits in melanoma cells; notably, an opposite effect was found after a long-term treatment of the cells with the Notch pathway inhibitor [355]. Thus, further studies are needed to confirm the role of the Notch signaling pathway as an effective molecular target to suppress the CSC subpopulation and to reverse drug resistance in melanoma.

The potential of targeting the HH/Gli pathway with anticancer agents endowed with a specific activity against CSCs has been recently explored in melanoma [351]. Specifically, Smo inhibitors (vismodegib and sonidegib) have been developed and clinically investigated; however, their use was limited due to the occurrence of resistance and serious side effects and to a low selectivity against CSCs [356,357]. Novel and more potent Smo inhibitors, based on acylguanidine scaffolds, have been recently developed and investigated for their potential anticancer activity. Pietrobono and coworkers demonstrated that, in melanoma cells, these compounds specifically target the Smo protein, thus leading to a decreased expression of Gli1, and significantly impair the self-renewal of the CSC subpopulation [358]. Ornidazole is an antibiotic used for the treatment of many bacterial and parasitic infections and was recently reported to suppress CD133+ melanoma CSCs by inhibiting the HH signaling pathway and hindering tumor growth in mouse models [359]. Together, these studies provide the basis for novel, HH/Gli-targeted therapeutic approaches for the eradication of CSCs in melanoma.

So far, different compounds have been developed and reported to impair drug resistance in cancer cells endowed with stem-like traits, including melanoma CSCs, through the suppression of the PI3K/Akt/mTOR signaling cascade. Some of these inhibitors have been approved by the FDA and are presently being investigated in preclinical studies as well as in clinical trials [360,361,362,363,364]. Recently, it has been demonstrated that evodiamine, a novel alkaloid isolated from the fruit of tetradium, significantly inhibits the growth of vemurafenib-resistant melanoma cells by inhibiting the PI3K/Akt signaling pathway through the suppression of IRS4 (insulin receptor substrate 4) [364]. The anaplastic lymphoma kinase (ALK) transcript has been shown to be overexpressed in melanoma cells and to induce CSC-like properties by promoting sphere formation and upregulating the expression of stem cell markers [365]; TAE684, a pharmacological inhibitor of ALK, in combination with a PI3K/mTOR inhibitor, reverts the stemness properties of these cells [360]. Itraconazole, a commonly used antifungal compound, has been observed to impair the growth and colony-formation ability of melanoma cells by hampering the functions of the PI3K/mTOR, HH and Wnt intracellular signaling pathways [361]. Corrales and colleagues described that the PI3K/Akt pathway is overactivated in melanoma cells made resistant to MAPK/ERK inhibitors (vemurafenib or trametinib) and is associated with increasing cell quiescence and migratory properties; notably, treatment of these cells with the dual PI3K/mTOR inhibitor dactolisib markedly reverts their stem-like properties [207]. Thus, impairing the activity of the PI3K/Akt/mTOR intracellular signaling cascade might represent a promising strategy for advancing CSC-targeted therapies for melanoma treatment.

**Table 1:** Therapeutic agents targeting melanoma CSC markers.

Markers	Agents	Actions	References
CD271	Anti-CD271 mAb	Depletion of CD271+ cells and suppression of tumor growth (in vitro and in vivo studies)	[101]
	CD271S + anti-CD47 mAb	Inhibition of tumor metastasis in patient-derived xenografts	[94]
	Aβ(25–35) + chemotherapy or targeted therapy	Activation of CD271 intracellular domain; induction of apoptosis and reduced tumor volume and metastasis; drug resistance overcome	[330]
CD133	Anti-CD133 mAb	Cytotoxic effects	[331]
	CD133 shRNA	Reduced metastatic behavior (in vitro and in vivo studies)	[331]
	AC133-saporin	Decreased cell proliferation and colony-forming ability	[332]
CD20	Rituximab	Regression of tumor growth in patients	[333]
	Rituximab + anti-PD-1 mAb	Beneficial toxicity profile	[334]
	Rituximab + BRAF inhibitor	Potentiation of vemurafenib-mediated cell killing	[335]
	VCR-Lip-CD20	Decreased ability of melanosphere formation	[336]
	CD20-SA-NPs	Cytotoxic activity and decreased melanosphere formation	[339]
	ACEXO	Decreased melanosphere formation and reduced tumor growth in nude mice	[340]
CD44	PTX-loaded HA-SLNs	Suppression of CD44+ cells	[341]
	HA-eNPs/ATRA	Decreased tumorigenicity and metastatic potential	[342]
	RG7356 (anti-CD44 mAb)	Good toleration, low clinical efficacy in patients	[343]
ABCB5	Anti-ABCB5 mAb	Reversion of drug resistance and increased sensitivity to standard treatments	[84]
ABCG2	δ-Tocotrienol	Decreased ability of melanosphere formation, promotion of melanosphere disaggregation	[78]
ALDH	Nifuroxazide	Suppression of ALDH+ stemness features	[131]
	AGI-101H	Induction of cytotoxic T cells specific for ALDH1A1	[344]
	ALDH1A1 + ALDH1A3 dual peptide–dendritic cell vaccine	Induction of T cell immunity against ALDH+ cells	[133]
Stemness-associated transcription factors (Sox2, Nanog, Oct4)	Triphenylmethane gentian violet, lunasin, retinoid acid, chelerythrine chloride	Decreased CSC self-renewal, reduced colony formation and tumor growth in mice, CSC differentiation, inhibition of sphere formation	[345,346,347,348]

**Table 2:** Therapeutic agents targeting melanoma CSC signaling pathways.

Signaling Pathways	Agents	Actions	References
Wnt/β-catenin	Pimozide	Inhibition of CSC features through the downregulation of the STAT-3 and 5 pathway	[169]
	35b	Reduced melanosphere formation and ALDH expression	[168]
	Morin	Inhibition of cell proliferation, self-renewal and melanosphere formation	[350]
Notch	RO4929097, honokiol	Decreased melanoma cell proliferation and ability to acquire stemness features	[353,354]
Hedgehog/Gli	Acylguanidine derivatives	Inhibition of self-renewal properties	[358]
	Ornidazole	Induced DNA damage in CD133+ melanoma cells and suppression of tumor growth in vivo	[359]
PI3K/Akt/mTOR	Evodiamine	Inhibition of the growth of vemurafenib-resistant cells through the suppression of IRS4	[364]
	TAE684 + PI3K/mTOR inhibitor	Reversion of stemness properties	[360]
	Itraconazole	Decreased colony formation ability	[361]
	Dactolisib	Reversion of stemness properties in targeted therapy-resistant cells	[207]

## 6. Conclusions and Future Directions

Cancer cell heterogeneity and plasticity represent a peculiar feature of most solid tumors, including melanoma. In this context, CSCs are a subpopulation of slowly replicating cells endowed with the capacities of cell renewal, proliferation and differentiation, giving rise to the bulk tumor mass; they are also deeply involved in the processes of tumor progression, drug resistance and relapse. Thus, elucidating their molecular and biological features might pave the way for developing novel therapeutic strategies for melanoma patients. From the molecular point of view, melanoma CSCs are defined by the presence of specific surface markers, drug efflux transporters, intracellular markers, pluripotency-associated transcription factors and activated signaling pathways. During the last few years, significant efforts have been made to dissect the molecular mechanisms underlying the biological functions of melanoma CSCs, particularly their role in the development of drug resistance. Accumulated evidence has pointed out an intricate interplay between CSCs and their neighboring cells in the TME. Specifically, it has been reported that melanoma CSCs possess remarkable immune evasion capacities and the ability to selectively recruit and activate immune cells endowed with both immunosuppressive and protumor properties. Moreover, melanoma CSCs release biofactors that improve neoangiogenesis and can differentiate into endothelial-like cells, thus favoring new vessel formation (VM); on the other hand, endothelial cells were reported to sustain CSC plasticity and promote their VM capacity. Notably, detrimental bidirectional crosstalk has also been detected between melanoma CSCs and CAFs. Finally, CSCs were found to horizontally transfer their stemness and aggressive features to bystander non-CSCs within the melanoma tumor mass. Together, these observations support that a precise eradication of CSCs, in a balanced combination with conventionally used therapies, might represent a new promising strategy for the management of melanoma patients, specifically in the advanced and drug-resistant stage. Different compounds targeting CSC molecular and biological traits have been developed and their efficacy in CSC killing has been reported.

However, some challenges remain to be addressed. Firstly, a thorough clarification of the mechanisms involved in the reciprocal CSC-TME crosstalk is still incomplete. Moreover, most of the results so far reported on the activity of CSC-targeting agents derive from in vitro and preclinical studies. Thus, clinical trials confirming these promising observations are urgently needed.

## Data Availability

No new data were created or analyzed in this study. Data sharing is not applicable to this article.
